# The forbidden touch: mechanical clearing of gas-induced crystalline lens feathering during vitrectomy surgery

**DOI:** 10.1186/s40942-024-00565-1

**Published:** 2024-07-22

**Authors:** Murtaza M. Mandviwala, Matthew K. Adams, Andrew J. Barkmeier

**Affiliations:** 1 Department of Ophthalmology, Mayo Clinic, 200 1st St SW, Rochester, MN 55905 USA; 2 Texas Macula and Retina, Plano, TX USA

## Abstract

**Background and objective:**

Lens feathering due to intraocular gas may cause significant challenges with intraoperative visualization during posterior segment surgery. Herein, we describe an intraoperative technique for improving posterior segment visualization impacted by lens feathering.

**Methods:**

New technique to improve visualization in vitrectomy from gas-induced cataract.

**Results:**

The light pipe is used to gently massage posterior subcapsular lens vacuoles to improve the surgical view intraoperatively.

**Conclusion:**

We report an effective and efficient technique to improve lens feathering during vitreoretinal surgery without need for cataract extraction.

**Supplementary Information:**

The online version contains supplementary material available at 10.1186/s40942-024-00565-1.

## Introduction

We present a novel technique for managing visually significant intraocular gas-related crystalline lens feathering during vitreoretinal surgery. Lens feathering, though not fully understood, is believed to result from either oxidation or the desiccation of the posterior lens surface upon contact with intraocular gas, potentially disrupting the epithelial layer’s metabolism and giving rise to transient subcapsular lens opacities. [1, 2] This phenomenon can hamper both diagnostic evaluation of the posterior segment and surgical visualization during vitreoretinal procedures. In this report, we outline an intraoperative technique utilizing the light pipe to delicately massage out transient gas-induced posterior lens vacuoles to significantly improve the posterior surgical view during vitrectomy procedures without the need for pars plana lensectomy.

### Technique

The procedure begins by inserting the light pipe through a cannula into the mid-vitreous cavity. The shaft of the light pipe is then used to gently massage the posterior capsule and flatten and displace the subcapsular vacuoles away from the central axis into the lens periphery (see Video 1). This process may be repeated in each quadrant as necessary; the infusion line can also be temporarily repositioned to another cannula to allow light pipe access through the inferotemporal port. Care must be taken to avoid inadvertent violation or aggressive manipulation of the posterior capsule, which could complicate future cataract extraction procedures. This risk can be mitigated by making sure only the shaft of the light pipe is in contact with the posterior capsule, rather than the sharper instrument tip. It is important to note that this technique is specifically applicable to transient lens feathering induced by air or gas, rather than other types of more chronic posterior subcapsular cataract.

## Discussion

Our patient initially underwent 23-gauge pars plana vitrectomy and membrane peeling with 14% C3F8 gas for fovea-involving combined tractional/rhegmatogenous retinal detachment due to proliferative diabetic retinopathy. Twenty-nine days later, a recurrent fovea-sparing retinal detachment was noted with significant gas-induced “feathering” cataract for which she underwent repeat 23-gauge vitrectomy with retinectomy, silicone oil, and massage of posterior capsule as described above. She underwent uncomplicated cataract surgery with intraocular lens placement in the capsular bag three months after the retinal detachment repair. She achieved final best visual acuity of 20/25 one year later. We have performed this technique on two other cases without complication.

When urgent or semi-urgent vitreoretinal surgery is required in the early post-operative period following a vitreoretinal procedure that utilized intraocular gas, surgical visualization may be significantly reduced if residual gas has induced posterior crystalline lens feathering. Depending on the degree of lens opacity, this may require either delaying surgery, proceeding with suboptimal visualization, or performing combined cataract surgery either via phacoemulsification or pars plana lensectomy. Intraocular lens calculations are often unavailable or, if obtained, less accurate in this situation due to persistent gas. If a combined scleral buckle procedure is indicated, the accuracy of pre-operative IOL calculations is reduced further. Although it is possible that this maneuver may risk posterior capsular rupture and/or accelerate cataract formation, assuming these risks may be offset by the potential benefits for select patients. We are unaware of previous methods of improving lens feathering during vitrectomy surgery without performing combined lensectomy or phacoemulsification. This technique allows for improved visualization of the posterior segment, vitreoretinal surgical efficiency, and optimizes refractive outcomes for patients who may have accurate IOL calculations and standard phacoemulsification procedures once their retinal status has stabilized.

### Electronic supplementary material

Below is the link to the electronic supplementary material.

Supplementary Material 1

## Data Availability

No datasets were generated or analysed during the current study.
